# Supplementary material for: Engineered transfer RNAs for suppression of premature termination codons
Source: Nat Commun. 2019 Feb 18;10:822. doi: 10.1038/s41467-019-08329-4 (PMC6379413; doi:10.1038/s41467-019-08329-4)
Supplement: Supplementary file 1 — Supplementary Information [file 41467_2019_8329_MOESM1_ESM.pdf]

# Engineered transfer RNAs for suppression of premature termination codons.

Lueck et al

| Amino Acid | | | Codons | | | | | |
|---|---|---|---|---|---|---|---|---|
| W | Trp | Tryptophan | **TGG** | | | | | |
| Y | Tyr | Tyrosine | **TAC** | **TAT** | | | | |
| C | Cys | Cysteine | **TGC** | **TGT** | | | | |
| E | Glu | Glutamic acid | **GAA** | **GAG** | | | | |
| K | Lys | Lysine | **AAA** | **AAG** | | | | |
| Q | Gln | Glutamine | **CAA** | **CAG** | | | | |
| S | Ser | Serine | AGC | AGT | **TCA** | TCC | **TCG** | TCT |
| L | Leu | Leucine | **TTA** | **TTG** | CTA | CTC | CTG | CTT |
| R | Arg | Arginine | **AGA** | AGG | **CGA** | CGC | CGG | CGT |
| G | Gly | Glycine | **GGA** | GGC | GGG | GGT | | |
| F | Phe | Phenylalanine | TTC | TTT | | | | |
| D | Asp | Aspartic acid | GAC | GAT | | | | |
| H | His | Histidine | CAC | CAT | | | | |
| N | Asn | Asparagine | AAC | AAT | | | | |
| M | Met | Methionine | ATG | | | | | |
| A | Ala | Alanine | GCA | GCC | GCG | GCT | | |
| P | Pro | Proline | CCA | CCC | CCG | CCT | | |
| T | Thr | Threonine | ACA | ACC | ACG | ACT | | |
| V | Val | Valine | GTA | GTC | GTG | GTT | | |
| I | Ile | Isoleucine | ATA | ATC | ATT | | | |
| X | STP | Stop codon | TAA | TAG | TGA | | | |

**Codon usage for common PTC.** Red highlight indicates the most common codons and corresponding amino acid type which can be converted to stop codons by a single nucleotide substitution. Engineered tRNAs have been developed for each type.

Supplementary Fig.1

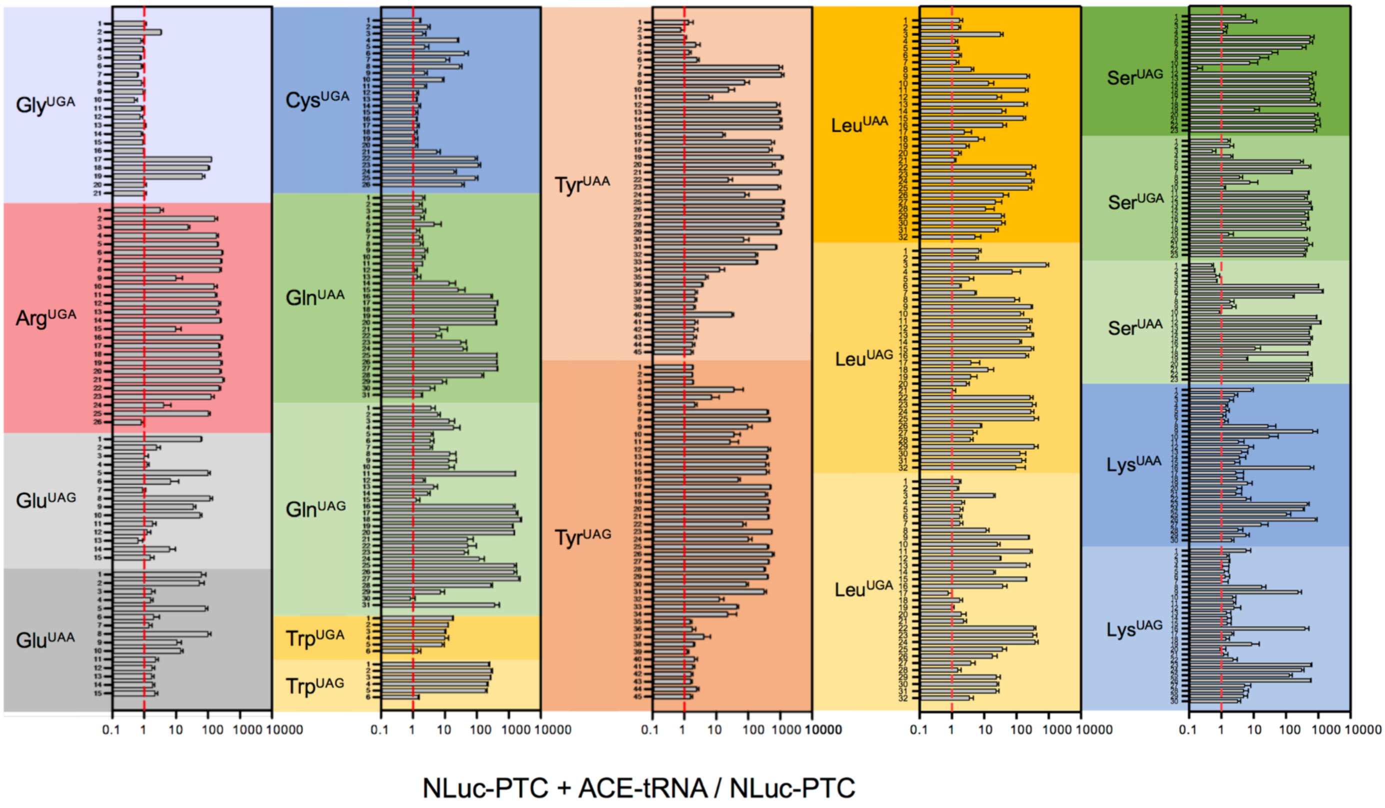


**Number referenced ACE-tRNA activity plot.**

Supplementary Fig.2A

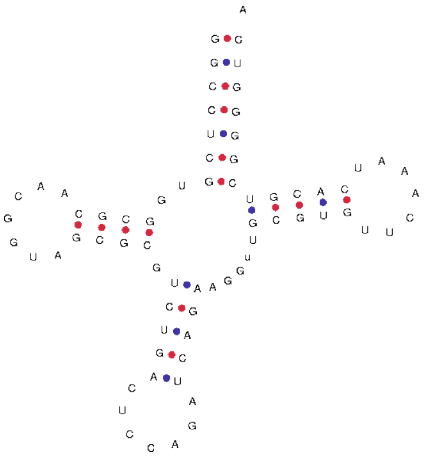

Trp-chr17.trna39
UGA and UAG

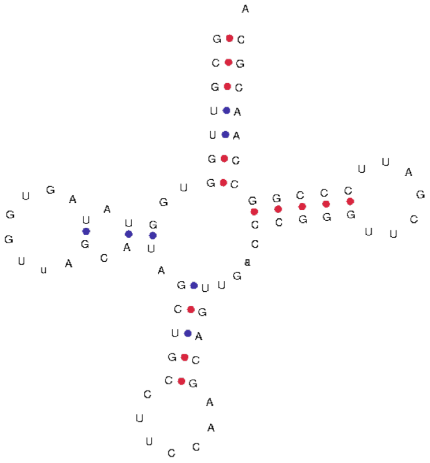

Gly-chr19.trna2
UGA

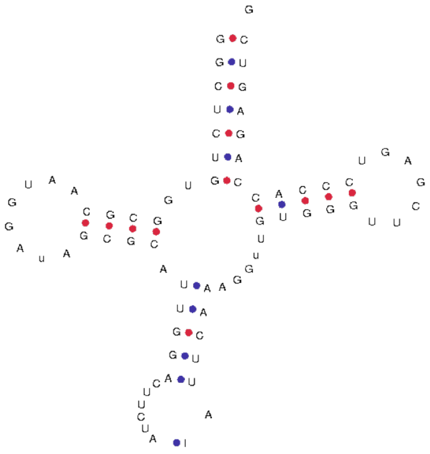

Arg-chr9.trna6
UGA

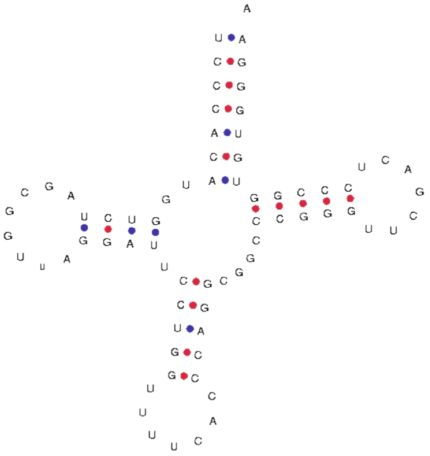

Glu-chr13.trna2
UAG and UAA

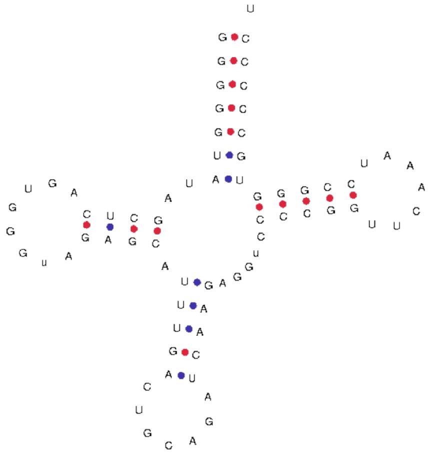

Cys-chr15.trna3
UGA

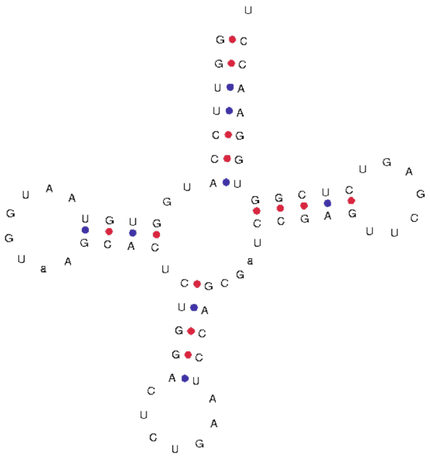

Gln-chr1.trna101
UAA not UAG

Supplementary Fig. 2B

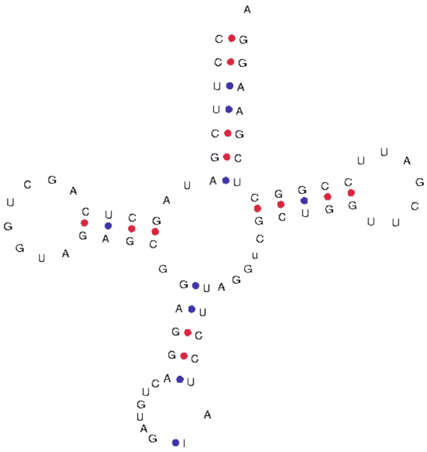

Tyr-chr14.trna17
UAA not UAG

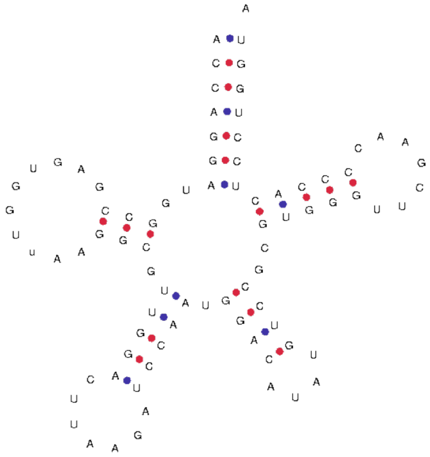

Leu-chr6.trna81
UGA and UAA, not UAG

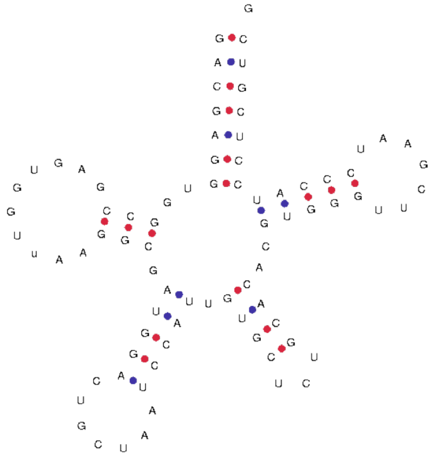

Ser-chr6.trna61

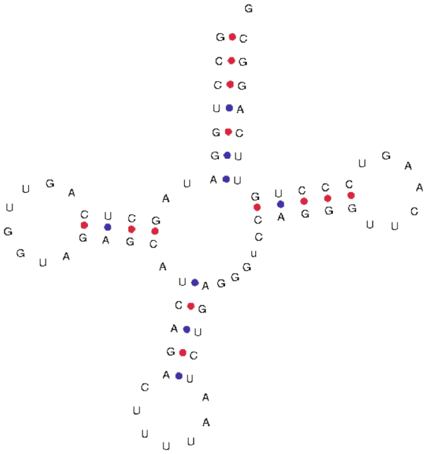

Lys-chr11.trna11
UAG not UAA

**Predicted cloverleaf structures for ACE-tRNA examples used in this study.** Anticodon type noted for versions that were functional for suppression. Cloverleaf predictions were based off the parent sequence (non-mutated) of each ACE-tRNA using tRNAscan-SE (http://lowelab.ucsc.edu/tRNAscan-SE/).

Supplementary Fig. 2B continued

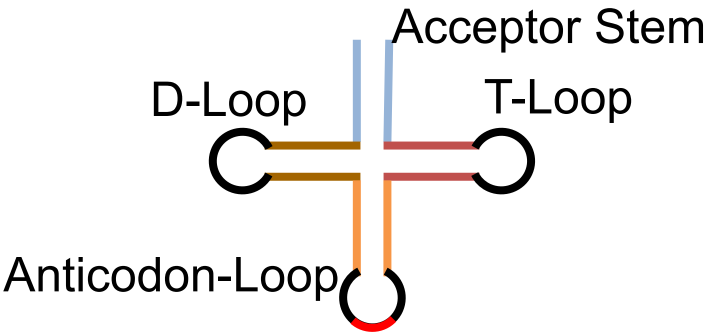


```
                                 >>>>>>>..>>>>.......<<<<.>>>>>.......<<<<<.....>>>>>.......<<<<<<<<<<<<.

1)  Glychr1.trna122         GCAUUGGUGGUUCAGUGGUAGAAUUCUCGCCUUCAACGCGGGAG-ACCCGGGUUCAAUUCCCGGCCAAUGCA
2)  Glychr2.trna25          GCGCCGCUGGUGUAGUGGUAUCAUGCAAGAUUUCAAUUCUUGCG-ACCCGGGUUCGAUUCCCGGGCGGCGCA
3)  Glychr17.trna11         GCAUUGGUGGUUCAAUGGUAGAAUUCUCGCCUUCAACGCAGGAG-ACCCAGGUUCGAUUCCUGGCCAAUGCA
4)  Glychr1.trna120         GCGUUGGUGGUUUAGUGGUAGAAUUCUCGCCUUCAAUGCGGGA-GACCCGGGUUCAAUUCCCGGCCACUGCA
5)  Glychr1.trna2           GCCUUGGUGGUGCAGUGGUAGAAUUCUCGCCUUCAACGUGGGAG-ACCCGGGUUCAAUUCCCGGCCAAUGCA
6)  Glychr1.trna83          GG-----UGGUUCAGUGGU-AGAAUUCUCGCCUUCAACGCGGGAGACCCGGGUUUAAUUCCCGG-----UCA
7)  Glychr2.trna1           G------UGGUCUAGUGGUUAGGAUUCAGCGCUUCAACCGCCGCAGCCCGGGUUCGAUUCCCGGU-----CA
8)  Glychr1.trna2*          GCGUCAGUGGUUUAGUGGUGGAAUUCCUGCCUUCAAUGCACGAG-AUCCGUGUUCAACUCCUGGUUGGUGCA
9)  Glychr1.trna102         GCGUCAGUGGUUUUAGUGGUGGAAUUCCUGCCUUCAAUGCACGAGAUCCGUGUUCAACUCCUGGUUGGUGCA
10) Glychr1.trna16          GCGUUGGCAGUUCAGUGGUAGAAUUCUCGCCUUCAACCCGGGAG-ACCUGGAUUCCAUUUCCGGCAAAUGCA
11) Glychr1.trna34          GCAUGGGUGGUUCAGUGGUAGAAUUCUCGCCUUCAACGCGGGAG-GCCCGGGUUCGAUUCCCGGCCCAUGCA
12) Glychr1.trna61          GCAUUGGUGGUUCAGUGGUAGAAUUCUCGCCUUCAACGCGGGAG-GCCCGGGUUCGAUUCCCGGCCAAUGCA
13) Glychr16.trna25         GCAUUGGUGGUUCAGUGGUAGAAUUCUCGCCUUCAACGCGGGAG-GCCCGGGUUUGAUUCCCGGCCAGUGCA
14) Glychr1.trna42          GCAUAGGUGGUUCAGUGGUAGAAUUCUUGCCUUCAACGCAGGAG-GCCCAGGUUUGAUUCCUGGCCCAUGCA
15) Glychr16.trna19         GCAUUGGUGGUUCAGUGGUAGAAUUCUCGCCUUCAAUGCGGGCG-GCCGGGCUUCGAUUCCUGGCCAAUGCA
16) Glychr6.trna80          GCAUGGGUGAUUCAGUGGUAGAAUUUUCACCUUCAAUGCAGGAG-GUCCAGGUUCAUUUCCUGGCCUAUGCA
17) Glychr19.trna2          GCGUUGGUGGUAUAGUGGUUAGCAUAGCUGCCUUCAAAGCAGUUGACCCGGGUUCGAUUCCCGGCCAACGCA
18) Glychr1.trna107         GCGUUGGUGGUAUAGUGGUGAGCAUAGCUGCCUUCAAAGCAGUUGACCCGGGUUCGAUUCCCGGCCAACGCA
19) Glychr17.trna9          GCGUUGGUGGUAUAGUGGUAAGCAUAGCUGCCUUCAAAGCAGUUGACCCGGGUUCGAUUCCCGGCCAACGCA
20) Glychr1.trna75          GCGUUGGUGGUAUAGUGGUGAGCAUAGUUGCCUUCAAAGCAGUUGACCCGGGCUCGAUUCCCGCCCAACGCA
21) Glychr1.trna75-65C-G    GCGUUGGUGGUAUAGUGGUGAGCAUAGUUGCCUUCAAAGCAGUUGACCCGGGCUCGAUUCCCGGCCAACGCA
```

**Alignment of Glycine tRNA sequences.** 21 tRNA$^{Gly}$ human sequences demonstrate high sequence homology amongst tRNA clades. Color coding indicated in tRNA image.

Supplementary Fig. 3

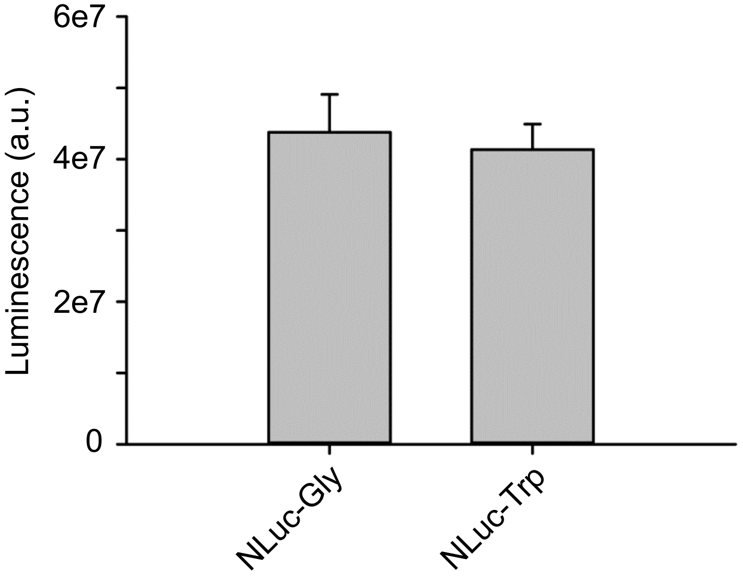


Side-chain identity at p.162 in Nanoluciferase does not effect activity. Total luminescence activity is indicated for each mutation at site.

Supplementary Fig. 4

**a**

**b**

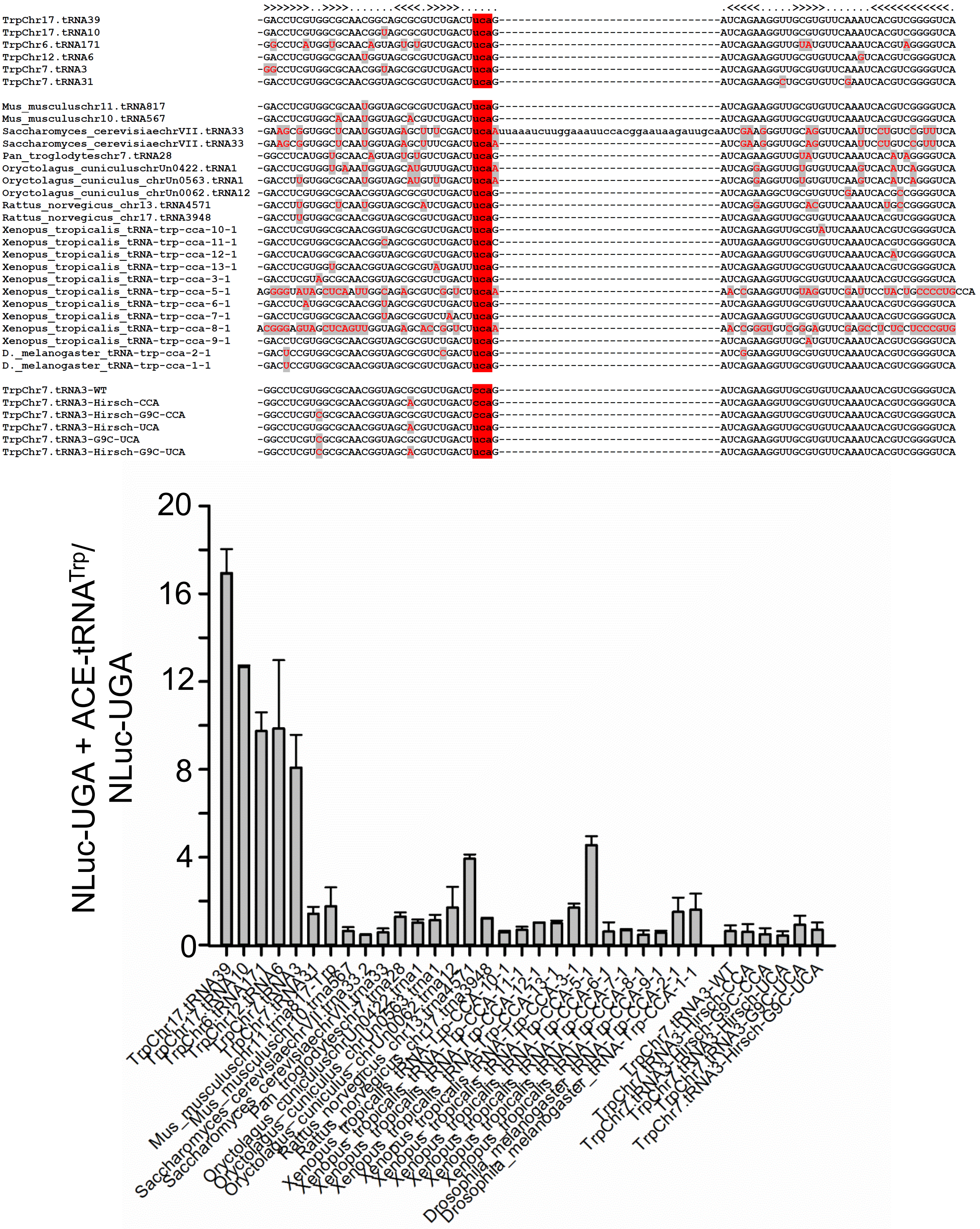


**Analysis of ACE-tRNA$^{Trp}$ sequences from multiple species and suppressor tRNA mutations.**

Supplementary Fig. 5

**a**

MSFNTIIDWNSCTAE**QQRQLLMRPAISASESITRTVNDILDNV**
**KARGDEALREYSAKFDKTTVTALKVSAEEIAAASERLSDELKQ**
**AMAVAVK*N**IETFHTAQK*LPPVDVETQPGVRCQQVTR**PVASVGL
YIPGGSAPLFSTVLMLATPASIAGCKKVVLCSPPPIADEILYA
AQLCGVQDVFNVGGAQAIAALAFGTESVPK**VDKIFGPGNAFVT**
**EAKR**QVSQR**LDGAAIDMPAGPSEVLVIADSGATPDFVASDLLS**
**QAEHGPDSQVILLTPAADMARRVAEAVERQLAELPR**AETARQ**A**
**LNASR**LIVTK**DLAQCVEISNQYGPEHLIIQTR**NAR**ELVDSITS**
**AGSVFLGDWSPESAGDYASGTNHVLPTYGYTATCSSLGLADFQ**
**KRMTVQELSKEGFSALASTIETLAAAERLTAHKNAVTLR**VNAL
KEQA**HHHHHHHHS*GGSAWSHPQFEK***

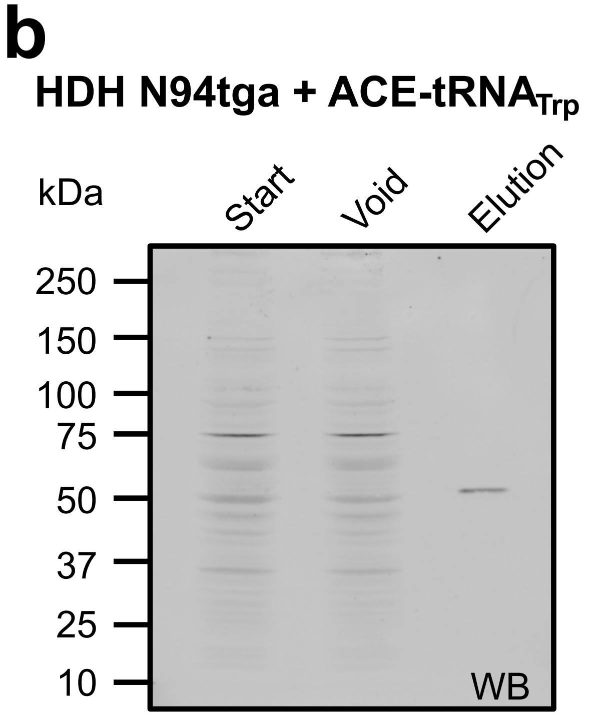


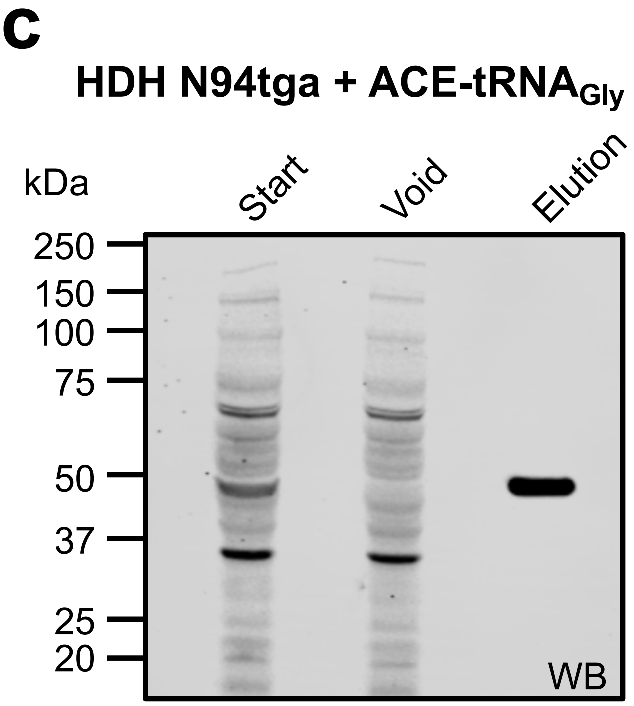


**Histidinol dehydrogenase (HDH) His(8)-streptactin expression construct allows for efficient one-step isolation of protein from HEK293 cells. a)** Protein sequence of HDH expression construct. Underlined sequence indicates coverage by mass spectrometry. The red asparagine (a.a. position 94) is the residue mutated to a TGA PTC for determining ACE-tRNA fidelity. The dual affinity tag is indicated in green. Silver stain of HDH protein following PTC suppression with **b)** Trpchr17.trna39 and **c)** Glychr19.trna2 .

Supplementary Fig. 6

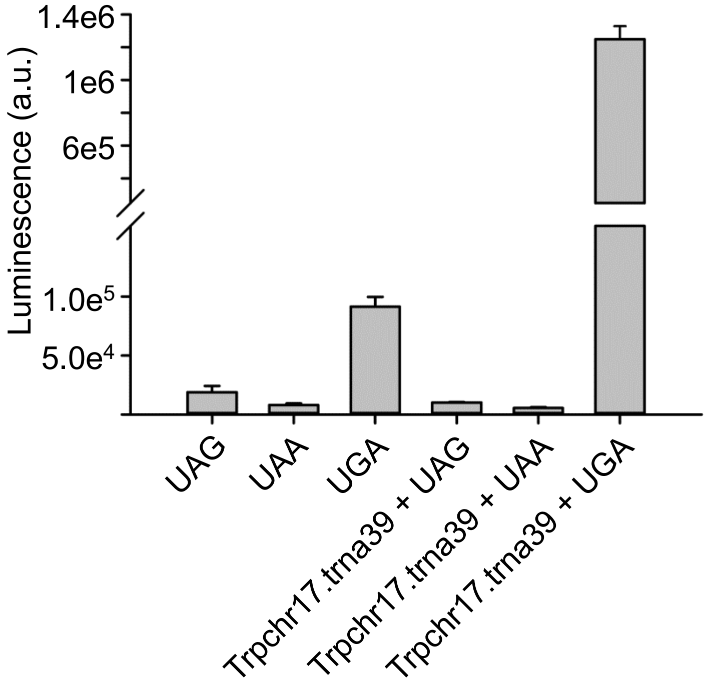


**Stop codon specificity is maintained for ACE-tRNA$^{Trp}$**. Suppression activity for tRNA Trp$^{TGA}$ Trpchr17.trna39, the top performing Trp$^{TGA}$ suppressor tRNA, Figure 2. This tRNA was co-expressed with the indicated pNano-STOP plasmid.

Supplementary Fig. 7

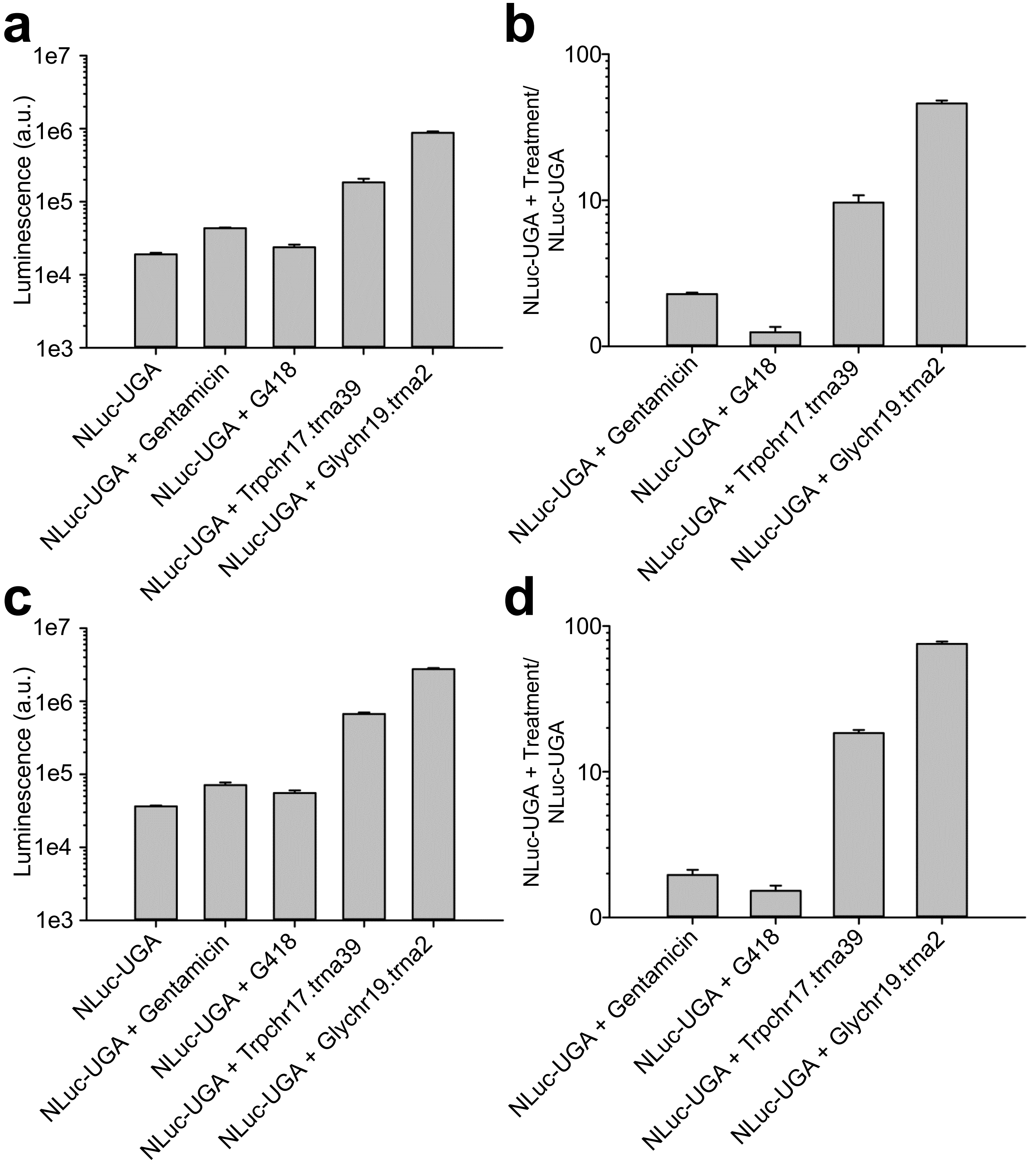


**ACE-tRNAs are more efficient than aminoglycoside PTC suppression. a)** Raw and **b)** normalized luminescence measured 24hrs following addition of gentamicin (40uM), G418 (150uM) and transfection with Trpchr17.trna39 and Glychr19.trna2 in HEK293 cells stably expressing PTC reporter Nluc-UGA. **c)** Raw and **d)** normalized luminescence measured 24hrs following addition of gentamicin (40uM), G418 (150uM) and co-transfection with Trpchr17.trna39 and Glychr19.trna2 in HEK293 cells.

Supplementary Fig. 8

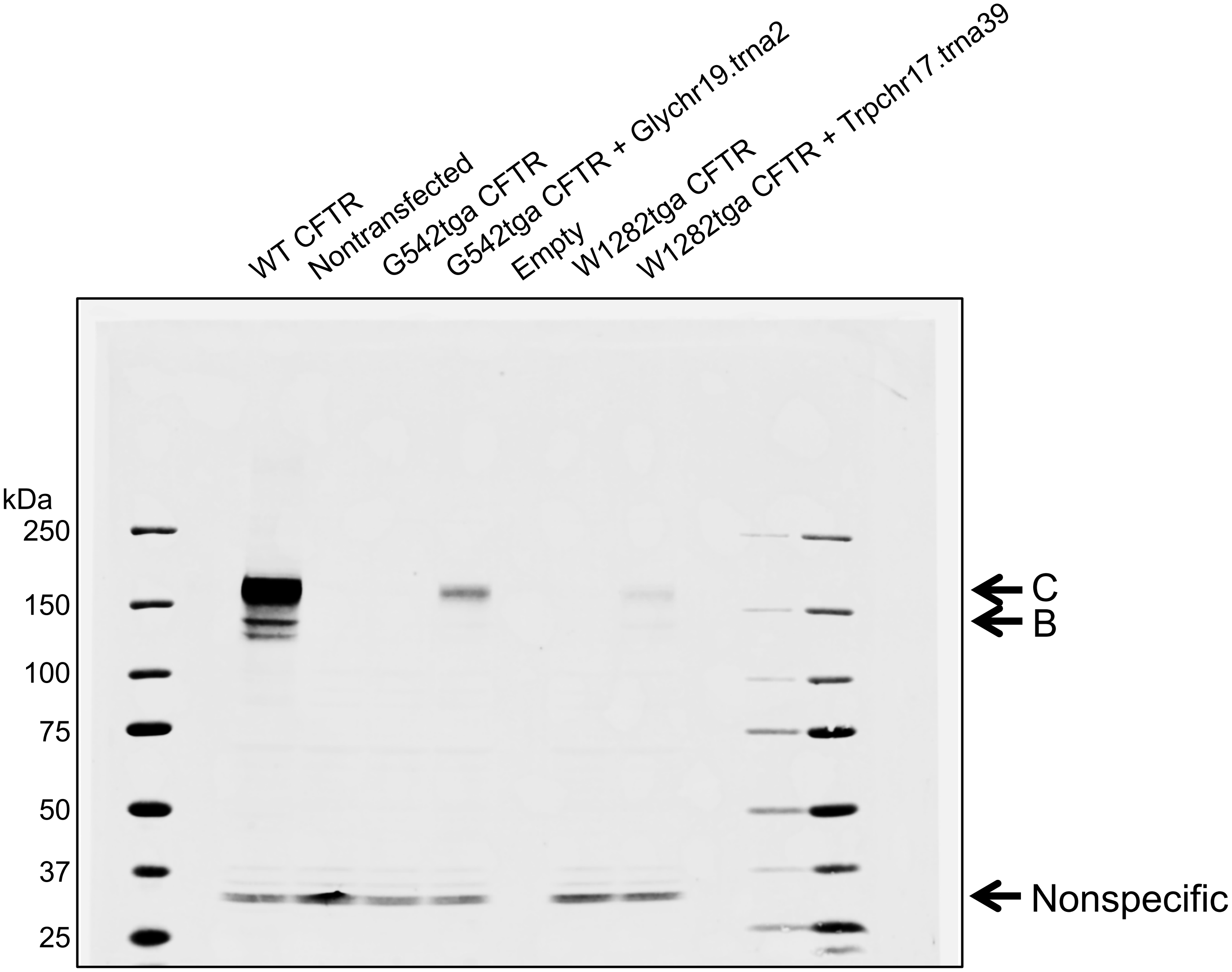


**Full-length Western blot figure 5d.**

Supplementary Fig. 9

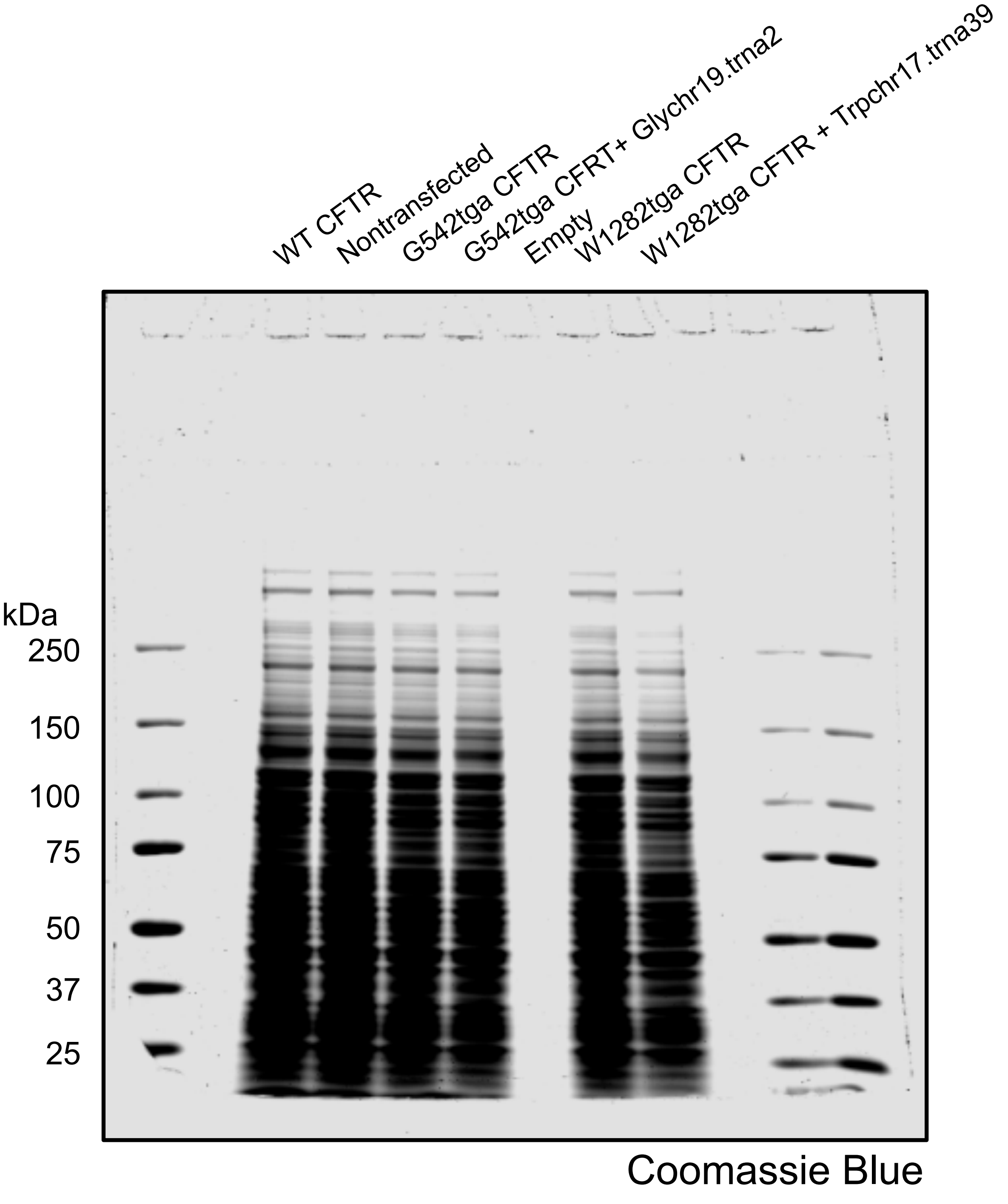


**Full-length Coomassie stain of replicate SDS-PAGE gel for figure 5d.**

Supplementary Fig. 10

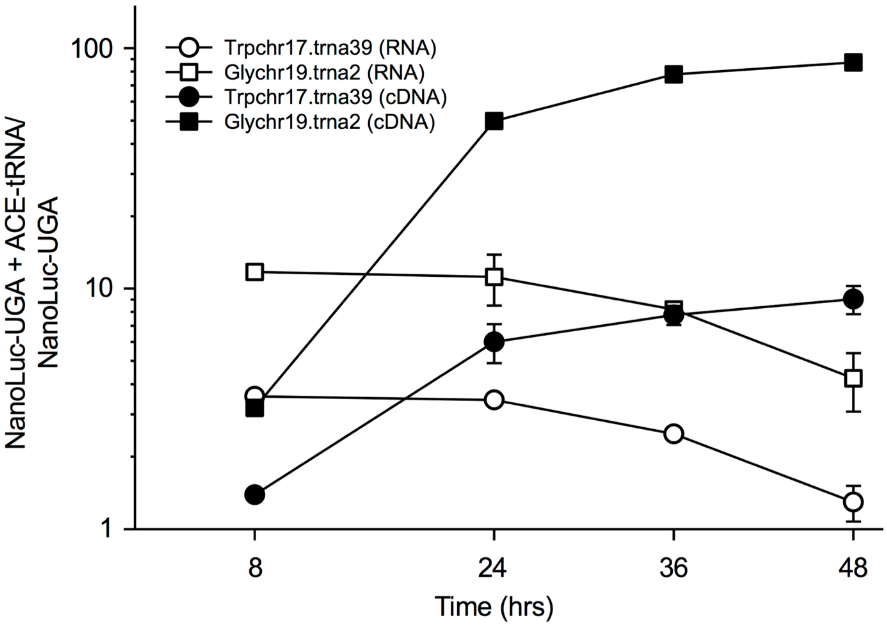


**Comparison of time courses of ACE-tRNA activity following delivery as RNA or cDNA.** ACE-tRNAs were delivered to HEK293 cells that stably express pNanoLuc-UGA as described in the methods, however only 5μl of the reaction mix was added to the cells to reduce the effect of transfection reagents on cell viability. ACE-tRNA delivered as RNA (open symbols), was more rapid in rescuing expression of the PTC reporter than cDNA constructs (closed circles). However, ACE-tRNA activity continued to rise over the 48 hrs when expressed from cDNA and decreased as a RNA deliverable.

Supplementary Fig. 11

**Supplementary Table 1.** rRNA depletion oligos

| Reference | Sequence (5’ to 3’) |
|---|---|
| NR_003285 | TCCTCCCGGGGCTACGCCTGTCTGAGCGTCGCT |
| NR_003286 | GGGGGGATGCGTGCATTTATCAGATCA |
| | TCCCCGCCCCTTGCCTCTCGGCGCCC |
| | TTGGTGACTCTAGATAACCTCGGGCCGATCGC |
| | GAGCCGCCTGGATACCGCAGCTAGGAATAATGGA |
| NR_003287 | TCGTGGGGGGCCCAAGTCCTTCTGATCGAGGCCC |
| | TCCGCCGAGGGCGCACCACCGGCCCGTCTCGCC |
| | GAGCCTCGGTTGGCCTCGGATAGCCGGTCCCCCGC |
| | GGGGCCGGGCCACCCCTCCCACGGCGCG |
| | GCGCCCCGGGCGGGTCGCGCCGTCGGGCCCGG |
| | CTCGCTTCTGGCGCCAAGCGCCCGGC |
| | GCGCGCCGCGGCTGGACGAGGCGCCGCCGCC |

Supplementary Table 2: Oligo Sequences (5'-3')

| | |
|---|---|
| | pNanoLuc plasmid for High-throughput cloning and screening mutagenesis primers. |
| pNanoLuc-For | GCCACCATGGTATTCACACTCGAAGATTTCGTTG |
| pNanoLuc- TGA-Rev | TTACGCCAGAATGCGTTCGCACAGCCGCCAGCCGGTCACTCATCC |
| pNanoLuc- TAA-Rev | TTACGCCAGAATGCGTTCGCACAGCCGCCAGCCGGTCACTTATCC |
| pNanoLuc- TAG-Rev | TTACGCCAGAATGCGTTCGCACAGCCGCCAGCCGGTCACCTATCC |
| pNanoLuc- WT-Rev | TTACGCCAGAATGCGTTCGCACAGCCGCCAGCCGGTCACTCCACC |
| | pNanoLuc plasmid for High-throughput cloning and screening Gibson assembly primers. |
| pNanoLuc-GAFor | ctggctagcgtttaaacttaagcttGCCACCATGGTATTCACAC |
| pNanoLuc- TGA-GARev | actgtgctggatatctgcagaattcTTACGCCAGAATGCGTTC |
| pNanoLuc- TAA-GARev | actgtgctggatatctgcagaattcCTACGCCAGAATGCGTTC |
| pNanoLuc- TAG-GARev | actgtgctggatatctgcagaattcTTACGCCAGAATGCGTTC |
| pNanoLuc- WT-GARev | actgtgctggatatctgcagaattcTTACGCCAGAATGCGTTC |
| | Primers for generation of 4 x ACE-tRNA expression Golden Gate puc57 plasmid |
| tRNA 1F | cacagacgaagactgttcctaatacgactcactatagagcg |
| tRNA 1R | cacagacgaagactggtattaaattaaccctcactaaagcaaa |
| tRNA 2F | cacagacgaagacgtatacgactcactatagagcg |
| tRNA 2R | cacagacgaagacgttcgtattaaattaaccctcactaaagcaaa |
| tRNA 3F | cacagacgaagacctacgactcactatagagcgc |
| tRNA 3R | cacagacgaagaccttaaccctcactaaagcaaaaaa |
| tRNA 4F | cacagacgaagacacgttaatttaatacgactcactatagagcg |
| tRNA 4R | cacagacgaagacaccctgaattaaccctcactaaagcaaa |
| | Oligos for Golden Gate MCS in puc57 (EcoRI/HindIII) |
| GoldenGateMCS-For | aattcttcccgagacgTTCCAAGTCTTCatGAAGACTACAGGcgtctcccagga |
| GoldenGateMCS-Rev | agcttcctgggagacgCCTGTAGTCTTCatGAAGACTTGGAAcgtctcgggaag |
| | Primers for amplification SV40 polyA termination signal and Gibson Assembly |
| SV40 term-For | acagtggcggccgctcgagtTTGTTTATTGCAGCTTATAATG |
| SV40 term-Rev | tggccgattcattaatgcagctgATAAGATACATTGATGAGTTTGG |
| | |

Synthesized ccdB Golden Gate cDNA (digested plasmid with PciI and Gibson assembled cDNA)

tcttatcatgtctggatcgaCTAGAGGGCCTTCCTAATACGACTCACTATAGAGCGCTCCGGTTTTTCTG
TGCTGAACCTCAGGGGACGCCGACACACGTACACGTCTAGTCTTCGCGGCCGCATTAGGCACCCCAGGCT
TTACACTTTATGCTTCCGGCTCGTATAATGTGTGGATTTTGAGTTAGGATCCGGCGAGATTTTCAGGAGC
TAAGGAAGCTAAAATGGAGAAAAAAATCACTGGATATACCACCGTTGATATATCCCAATGGCATCGTAAA
GAACATTTTGAGGCATTTCAGTCAGTTGCTCAATGTACCTATAACCAGACCGTTCAGCTGGATATTACGG
CCTTTTTAAAGACCGTAAAGAAAAATAAGCACAAGTTTTATCCGGCCTTTATTCACATTCTTGCCCGCCT
GATGAATGCTCATCCGGAATTCCGTATGGCAATGAAAGACGGTGAGCTGGTGATATGGGATAGTGTTCAC
CCTTGTTACACCGTTTTCCATGAGCAAACTGAAACGTTTTCATCGCTCTGGAGTGAATACCACGACGATT
TCCGGCAGTTTCTACACATATATTCGCAAGATGTGGCGTGTTACGGTGAAAACCTGGCCTATTTCCCTAA
AGGGTTTATTGAGAATATGTTTTTCGTCTCAGCCAATCCCTGGGTGAGTTTCACCAGTTTTGATTTAAAC
GTGGCCAATATGGACAACTTCTTCGCCCCCGTTTTCACCATGGGCAAATATTATACGCAAGGCGACAAGG
TGCTGATGCCGCTGGCGATTCAGGTTCATCATGCCGTCTGTGATGGCTTCCATGTCGGCAGAATGCTTAA
TGAATTACAACAGTACTGCGATGAGTGGCAGGGCGGGGCGTAAAGATCTGGATCCGGCTTACTAAAAGCC
AGATAACAGTATGCGTATTTGCGCGCTGATTTTTGCGGTATAAGAATATATACTGATATGTATACCCGAA
GTATGTCAAAAAGAGGTGTGCTATGAAGCAGCGTATTACAGTGACAGTTGACAGCGACAGCTATCAGTTG
CTCAAGGCATATATGATGTCAATATCTCCGGTCTGGTAAGCACAACCATGCAGAATGAAGCCCGTCGTCT
GCGTGCCGAACGCTGGAAAGCGGAAAATCAGGAAGGGATGGCTGAGGTCGCCCGGTTTATTGAAATGAAC
GGCTCTTTTGCTGACGAGAACAGGGACTGGTGAAATGCAGTTTAAGGTTTACACCTATAAAAGAGAGAGC
CGTTATCGTCTGTTTGTGGATGTACAGAGTGATATTATTGACACGCCCGGGCGACGGATGGTGATCCCCC
TGGCCAGTGCACGTCTGCTGTCAGATAAAGTCTCCCGTGAACTTTACCCGGTGGTGCATATCGGGGATGA
AAGCTGGCGCATGATGACCACCGATATGGCCAGTGTGCCGGTCTCCGTTATCGGGGAAGAAGTGGCTGAT
CTCAGCCACCGCGAAAATGACATCAAAAACGCCATTAACCTGATGTTCTGGGGAATATAAATGTCAGGCT
CCGTTATACACAGCCAGTCTGCAGGGAAgaagaccgGTCCTTTTTTTGCTTTAGTGAGGGTTAATTCAGT
GAGCAAAAGGC

The following mutants gibson assembled into the naturally occurring HindIII-EcoRI sites:

pNanoLuc-TGA
gccaccATGGTaTTCACACTCGAAGATTTCGTTGGGGACTGGCGACAGACAGCCGGCTACAACC
TGGACCAAGTCCTTGAACAGGGAGGTGTGTCCAGTTTGTTTCAGAATCTCGGGGTGTCCGTAAC
TCCGATCCAAAGGATTGTCCTGAGCGGTGAAAATGGGCTGAAGATCGACATCCATGTCATCATC
CCGTATGAAGGTCTGAGCGGCGACCAAATGGGCCAGATCGAAAAAATTTTTAAGGTGGTGTACC
CTGTGGATGATCATCACTTTAAGGTGATCCTGCACTATGGCACACTGGTAATCGACGGGGTTAC
GCCGAACATGATCGACTATTTCGGACGGCCGTATGAAGGCATCGCCGTGTTCGACGGCAAAAAG
ATCACTGTAACAGGGACCCTGTGGAACGGCAACAAAATTATCGACGAGCGCCTGATCAACCCCG
ACGGCTCCCTGCTGTTCCGAGTAACCATCAACGGATGAG**TGA**CCGGCTGGCGGCTGTGCGAACG
CATTCTGGCG*TAA*

pNanoLuc-TAA
gccaccATGGTaTTCACACTCGAAGATTTCGTTGGGGACTGGCGACAGACAGCCGGCTACAACC
TGGACCAAGTCCTTGAACAGGGAGGTGTGTCCAGTTTGTTTCAGAATCTCGGGGTGTCCGTAAC
TCCGATCCAAAGGATTGTCCTGAGCGGTGAAAATGGGCTGAAGATCGACATCCATGTCATCATC
CCGTATGAAGGTCTGAGCGGCGACCAAATGGGCCAGATCGAAAAAATTTTTAAGGTGGTGTACC
CTGTGGATGATCATCACTTTAAGGTGATCCTGCACTATGGCACACTGGTAATCGACGGGGTTAC
GCCGAACATGATCGACTATTTCGGACGGCCGTATGAAGGCATCGCCGTGTTCGACGGCAAAAAG

ATCACTGTAACAGGGACCCTGTGGAACGGCAACAAAATTATCGACGAGCGCCTGATCAACCCCG
ACGGCTCCCTGCTGTTCCGAGTAACCATCAACGGATGAG**TAA**CCGGCTGGCGGCTGTGCGAACG
CATTCTGGCG*TAG*

pNanoLuc-TAG
gccaccATGGTaTTCACACTCGAAGATTTCGTTGGGGACTGGCGACAGACAGCCGGCTACAACC
TGGACCAAGTCCTTGAACAGGGAGGTGTGTCCAGTTTGTTTCAGAATCTCGGGGTGTCCGTAAC
TCCGATCCAAAGGATTGTCCTGAGCGGTGAAAATGGGCTGAAGATCGACATCCATGTCATCATC
CCGTATGAAGGTCTGAGCGGCGACCAAATGGGCCAGATCGAAAAAATTTTTAAGGTGGTGTACC
CTGTGGATGATCATCACTTTAAGGTGATCCTGCACTATGGCACACTGGTAATCGACGGGGTTAC
GCCGAACATGATCGACTATTTCGGACGGCCGTATGAAGGCATCGCCGTGTTCGACGGCAAAAAG
ATCACTGTAACAGGGACCCTGTGGAACGGCAACAAAATTATCGACGAGCGCCTGATCAACCCCG
ACGGCTCCCTGCTGTTCCGAGTAACCATCAACGGATGAG**TAG**CCGGCTGGCGGCTGTGCGAACG
CATTCTGGCG*TAA*

pNanoLuc-WT
gccaccATGGTaTTCACACTCGAAGATTTCGTTGGGGACTGGCGACAGACAGCCGGCTACAACC
TGGACCAAGTCCTTGAACAGGGAGGTGTGTCCAGTTTGTTTCAGAATCTCGGGGTGTCCGTAAC
TCCGATCCAAAGGATTGTCCTGAGCGGTGAAAATGGGCTGAAGATCGACATCCATGTCATCATC
CCGTATGAAGGTCTGAGCGGCGACCAAATGGGCCAGATCGAAAAAATTTTTAAGGTGGTGTACC
CTGTGGATGATCATCACTTTAAGGTGATCCTGCACTATGGCACACTGGTAATCGACGGGGTTAC
GCCGAACATGATCGACTATTTCGGACGGCCGTATGAAGGCATCGCCGTGTTCGACGGCAAAAAG
ATCACTGTAACAGGGACCCTGTGGAACGGCAACAAAATTATCGACGAGCGCCTGATCAACCCCG
ACGGCTCCCTGCTGTTCCGAGTAACCATCAACGGATGAG**TGG**CCGGCTGGCGGCTGTGCGAACG
CATTCTGGCG*TAA*

HDH-TGA-HIS-STREP
ATGTCATTCAATACTATAATCGACTGGAACAGCTGCACTGCTGAGCAGCAAAGACAGCTGTTGATGAGAC
CAGCGATTTCCGCGTCCGAAAGTATCACAAGAACGGTGAATGATATACTTGATAACGTGAAAGCCAGAGG
CGACGAGGCGCTTCGAGAGTACAGCGCTAAATTTGACAAAACTACCGTAACGGCACTTAAAGTCTCTGCT
GAAGAAATCGCAGCCGCCAGCGAGCGGCTTTCTGACGAACTCAAGCAGGCGATGGCCGTTGCTGTAAAA**T**
**GAT**ATCGAAACTTTCCACACCGCTCAAAAATTGCCTCCGGTTGATGTAGAAACACAACCCGGAGTCCGCT
GCCAGCAAGTTACCCGGCCTGTAGCCTCAGTCGGATTGTATATTCCGGGTGGATCTGCGCCTCTGTTCAG
CACAGTCCTCATGCTGGCGACGCCCGCATCTATCGCTGGATGTAAGAAAGTCGTATTGTGTTCTCCTCCC
CCTATTGCAGATGAAATATTGTACGCCGCCCAACTGTGTGGTGTCCAAGACGTATTCAATGTGGGAGGCG
CTCAGGCTATAGCTGCTTTGGCATTTGGCACAGAGTCTGTCCCCAAGGTCGACAAGATCTTCGGTCCAGG
AAATGCGTTTGTCACTGAGGCAAAAAGACAGGTCTCACAGAGACTGGATGGGGCAGCCATCGACATGCCG
GCTGGCCCATCAGAAGTACTGGTGATTGCTGACAGTGGAGCTACCCCGGACTTCGTCGCCTCCGACCTCC
TGTCCCAAGCTGAGCATGGTCCAGATAGTCAGGTTATCCTGCTTACCCCCGCGGCTGATATGGCCAGGCG
CGTCGCAGAAGCGGTTGAGCGCCAACTGGCGGAACTCCCGCGGGCGGAGACTGCAAGGCAAGCTCTTAAC
GCGTCCAGGCTGATTGTAACAAAAGATCTGGCCCAATGTGTAGAAATCTCAAACCAGTACGGACCAGAGC
ACTTGATCATACAGACGCGAAACGCTCGGGAGCTTGTTGACAGTATCACTTCAGCGGGGTCTGTTTTTCT
TGGGGATTGGTCTCCAGAATCCGCGGGCGATTACGCATCCGGCACAAACCATGTTCTTCCTACATACGGC
TATACTGCCACTTGTTCTAGTCTTGGGCTTGCCGACTTCCAGAAGCGCATGACCGTTCAAGAACTGTCCA
AAGAAGGATTTTCAGCGCTTGCTTCTACAATAGAAACGCTCGCCGCAGCTGAGAGACTCACTGCCCATAA
GAACGCAGTAACACTGCGCGTCAACGCTCTCAAGGAGCAGGCGcaccaccatcaccatcatcatcacTCC
GGGGGCAGTGCATGGTCACACCCTCAGTTTGAGAAGTAA

Genbank sequence for pNanoLuc-TGA-pcDNA3.1

```
pNanoLuc-TGA-pcDNA3.1

LOCUS       NanLuc_pcDNA3.1_BbsI_le 5025 bp ds-DNA     circular     8-
DEC-2018

DEFINITION  .

FEATURES             Location/Qualifiers

     misc_feature    3140..3163
                     /label=""
                     /ApEinfo_revcolor=#faac61
                     /ApEinfo_fwdcolor=#faac61

     variation       3176..3187
                     /label="3' term signal"
                     /ApEinfo_revcolor=#c7b0e3
                     /ApEinfo_fwdcolor=#c7b0e3

     rep_origin      complement(3255..3874)
                     /label="pBR322_origin"
                     /ApEinfo_revcolor=#d6b295
                     /ApEinfo_fwdcolor=#d6b295

     gene            complement(4029..4889)
                     /label="Ampicillin"
                     /ApEinfo_revcolor=#b4abac
                     /ApEinfo_fwdcolor=#b4abac

     promoter        1739..1762
                     /label="lac_promoter"
                     /ApEinfo_revcolor=#f58a5e
                     /ApEinfo_fwdcolor=#f58a5e

     CDS             complement(4029..4889)
                     /label="ORF frame 2"
                     /ApEinfo_revcolor=#c7b0e3
                     /ApEinfo_fwdcolor=#c7b0e3
```

```
promoter        complement(4931..4959)
                /label="AmpR_promoter"
                /ApEinfo_revcolor=#f8d3a9
                /ApEinfo_fwdcolor=#f8d3a9
promoter        863..881
                /label="T7_promoter"
                /ApEinfo_revcolor=#c6c9d1
                /ApEinfo_fwdcolor=#c6c9d1
promoter        236..852
                /label="CMV_immearly_promoter"
                /ApEinfo_revcolor=#b1ff67
                /ApEinfo_fwdcolor=#b1ff67
variation       1634..1706
                /label="5' human Tyr-tRNA leader"
                /ApEinfo_revcolor=#d59687
                /ApEinfo_fwdcolor=#d59687
tRNA            3176..3187
                /label="tRNA tyr"
                /ApEinfo_revcolor=#b7e6d7
                /ApEinfo_fwdcolor=#b7e6d7
tRNA            1652..1706
                /label="tRNA tyr"
                /ApEinfo_revcolor=#85dae9
                /ApEinfo_fwdcolor=#85dae9
CDS             1823..2482
                /label="ORF frame 1"
                /ApEinfo_revcolor=#d6b295
                /ApEinfo_fwdcolor=#d6b295
misc_feature    315..602
                /label="CAG_enhancer"
```

```
                 /ApEinfo_revcolor=#b7e6d7
                 /ApEinfo_fwdcolor=#b7e6d7
misc_feature     2824..3129
                 /label="ccdB"
                 /ApEinfo_revcolor=#b4abac
                 /ApEinfo_fwdcolor=#b4abac
misc_feature     1407..3701
                 /label=""
                 /ApEinfo_revcolor=#ff9ccd
                 /ApEinfo_fwdcolor=#ff9ccd
misc_feature     769..789
                 /label="CMV_fwd_primer"
                 /ApEinfo_revcolor=#75c6a9
                 /ApEinfo_fwdcolor=#75c6a9
misc_feature     1715..1732
                 /label=""
                 /ApEinfo_revcolor=#faac61
                 /ApEinfo_fwdcolor=#faac61
exon             917..1441
                 /label="nanluc"
                 /ApEinfo_revcolor=#c7b0e3
                 /ApEinfo_fwdcolor=#c7b0e3
source           1..5025
                 /label="Zeo(+)"
                 /ApEinfo_revcolor=#ffef86
                 /ApEinfo_fwdcolor=#ffef86
gene             1823..2482
                 /label="CAT/CamR"
                 /ApEinfo_revcolor=#b7e6d7
                 /ApEinfo_fwdcolor=#b7e6d7
```

polyA_signal 1476..1619

/label=""

/ApEinfo_revcolor=#d59687

/ApEinfo_fwdcolor=#d59687

ORIGIN

1 GACGGATCGG GAGATCTCCC GATCCCCTAT GGTCGACTCT CAGTACAATC TGCTCTGATG

61 CCGCATAGTT AAGCCAGTAT CTGCTCCCTG CTTGTGTGTT GGAGGTCGCT GAGTAGTGCG

121 CGAGCAAAAT TTAAGCTACA ACAAGGCAAG GCTTGACCGA CAATTGCATG AAGAATCTGC

181 TTAGGGTTAG GCGTTTTGCG CTGCTTCGCG ATGTACGGGC CAGATATACG CGTTGACATT

241 GATTATTGAC TAGTTATTAA TAGTAATCAA TTACGGGGTC ATTAGTTCAT AGCCCATATA

301 TGGAGTTCCG CGTTACATAA CTTACGGTAA ATGGCCCGCC TGGCTGACCG CCCAACGACC

361 CCCGCCCATT GACGTCAATA ATGACGTATG TTCCCATAGT AACGCCAATA GGGACTTTCC

421 ATTGACGTCA ATGGGTGGAC TATTTACGGT AAACTGCCCA CTTGGCAGTA CATCAAGTGT

481 ATCATATGCC AAGTACGCCC CCTATTGACG TCAATGACGG TAAATGGCCC GCCTGGCATT

541 ATGCCCAGTA CATGACCTTA TGGGACTTTC CTACTTGGCA GTACATCTAC GTATTAGTCA

601 TCGCTATTAC CATGGTGATG CGGTTTTGGC AGTACATCAA TGGGCGTGGA TAGCGGTTTG

661 ACTCACGGGG ATTTCCAAGT CTCCACCCCA TTGACGTCAA TGGGAGTTTG TTTTGGCACC

721 AAAATCAACG GGACTTTCCA AAATGTCGTA ACAACTCCGC CCCATTGACG CAAATGGGCG

781 GTAGGCGTGT ACGGTGGGAG GTCTATATAA GCAGAGCTCT CTGGCTAACT AGAGAACCCA

841 CTGCTTACTG GCTTATCGAA ATTAATACGA CTCACTATAG GGAGACCCAA GCTGGCTAGC

901 GTTTAAACTT AAGCTTgcca ccATGGTaTT CACACTCGAA GATTTCGTTG GGGACTGGCG

961 ACAGACAGCC GGCTACAACC TGGACCAAGT CCTTGAACAG GGAGGTGTGT CCAGTTTGTT

1021 TCAGAATCTC GGGGTGTCCG TAACTCCGAT CCAAAGGATT GTCCTGAGCG GTGAAAATGG

1081 GCTGAAGATC GACATCCATG TCATCATCCC GTATGAAGGT CTGAGCGGCG ACCAAATGGG

1141 CCAGATCGAA AAAATTTTTA AGGTGGTGTA CCCTGTGGAT GATCATCACT TTAAGGTGAT

1201 CCTGCACTAT GGCACACTGG TAATCGACGG GGTTACGCCG AACATGATCG ACTATTTCGG

1261 ACGGCCGTAT GAAGGCATCG CCGTGTTCGA CGGCAAAAAG ATCACTGTAA CAGGGACCCT

1321 GTGGAACGGC AACAAAATTA TCGACGAGCG CCTGATCAAC CCCGACGGCT CCCTGCTGTT

1381 CCGAGTAACC ATCAACGGAT GAGTGACCGG CTGGCGGCTG TGCGAACGCA TTCTGGCGTA

1441 AGAATTCTGC AGATATCCAG CACAGTGGCG GCCGCtcgag TCTAGATTGT TTATTGCAGC

1501 TTATAATGGT TACAAATAAA GCAATAGCAT CACAAATTTC ACAAATAAAG CATTTTTTTC

1561 ACTGCATTCT AGTTGTGGTT TGTCCAAACT CATCAATGTA TCTTATCATG TCTGGATCgC

1621 TAGAGGGCCT TCCTAATACG ACTCACTATA GAGCGCTCCG GTTTTTCTGT GCTGAACCTC

1681 AGGGGACGCC GACACACGTA CACGTCTAGT CTTCGCGGCC GCATTAGGCA CCCCAGGCTT

1741 TACACTTTAT GCTTCCGGCT CGTATAATGT GTGGATTTTG AGTTAGGATC CGGCGAGATT

1801 TTCAGGAGCT AAGGAAGCTA AAATGGAGAA AAAAATCACT GGATATACCA CCGTTGATAT

1861 ATCCCAATGG CATCGTAAAG AACATTTTGA GGCATTTCAG TCAGTTGCTC AATGTACCTA

1921 TAACCAGACC GTTCAGCTGG ATATTACGGC CTTTTTAAAG ACCGTAAAGA AAAATAAGCA

1981 CAAGTTTTAT CCGGCCTTTA TTCACATTCT TGCCCGCCTG ATGAATGCTC ATCCGGAATT

2041 CCGTATGGCA ATGAAAGACG GTGAGCTGGT GATATGGGAT AGTGTTCACC CTTGTTACAC

2101 CGTTTTCCAT GAGCAAACTG AAACGTTTTC ATCGCTCTGG AGTGAATACC ACGACGATTT

2161 CCGGCAGTTT CTACACATAT ATTCGCAAGA TGTGGCGTGT TACGGTGAAA ACCTGGCCTA

2221 TTTCCCTAAA GGGTTTATTG AGAATATGTT TTTCGTCTCA GCCAATCCCT GGGTGAGTTT

2281 CACCAGTTTT GATTTAAACG TGGCCAATAT GGACAACTTC TTCGCCCCCG TTTTCACCAT

2341 GGGCAAATAT TATACGCAAG GCGACAAGGT GCTGATGCCG CTGGCGATTC AGGTTCATCA

2401 TGCCGTCTGT GATGGCTTCC ATGTCGGCAG AATGCTTAAT GAATTACAAC AGTACTGCGA

2461 TGAGTGGCAG GGCGGGGCGT AAAGATCTGG ATCCGGCTTA CTAAAAGCCA GATAACAGTA

2521 TGCGTATTTG CGCGCTGATT TTTGCGGTAT AAGAATATAT ACTGATATGT ATACCCGAAG

2581 TATGTCAAAA AGAGGTGTGC TATGAAGCAG CGTATTACAG TGACAGTTGA CAGCGACAGC

2641 TATCAGTTGC TCAAGGCATA TATGATGTCA ATATCTCCGG TCTGGTAAGC ACAACCATGC

2701 AGAATGAAGC CCGTCGTCTG CGTGCCGAAC GCTGGAAAGC GGAAAATCAG GAAGGGATGG

2761 CTGAGGTCGC CCGGTTTATT GAAATGAACG GCTCTTTTGC TGACGAGAAC AGGGACTGGT

2821 GAAATGCAGT TTAAGGTTTA CACCTATAAA AGAGAGAGCC GTTATCGTCT GTTTGTGGAT

2881 GTACAGAGTG ATATTATTGA CACGCCCGGG CGACGGATGG TGATCCCCCT GGCCAGTGCA

2941 CGTCTGCTGT CAGATAAAGT CTCCCGTGAA CTTTACCCGG TGGTGCATAT CGGGGATGAA

3001 AGCTGGCGCA TGATGACCAC CGATATGGCC AGTGTGCCGG TCTCCGTTAT CGGGGAAGAA

3061 GTGGCTGATC TCAGCCACCG CGAAAATGAC ATCAAAAACG CCATTAACCT GATGTTCTGG

3121 GGAATATAAA TGTCAGGCTC CGTTATACAC AGCCAGTCTG CAGGGAAgaa gaccgGTCCT

3181 TTTTTTGCTT TAGTGAGGGT TAATTCAGGC ATGTGAGCAA AAGGCCAGCA AAAGGCCAGG

3241 AACCGTAAAA AGGCCGCGTT GCTGGCGTTT TTCCATAGGC TCCGCCCCCC TGACGAGCAT

3301 CACAAAAATC GACGCTCAAG TCAGAGGTGG CGAAACCCGA CAGGACTATA AAGATACCAG

3361 GCGTTTCCCC CTGGAAGCTC CCTCGTGCGC TCTCCTGTTC CGACCCTGCC GCTTACCGGA

3421 TACCTGTCCG CCTTTCTCCC TTCGGGAAGC GTGGCGCTTT CTCAATGCTC ACGCTGTAGG

3481 TATCTCAGTT CGGTGTAGGT CGTTCGCTCC AAGCTGGGCT GTGTGCACGA ACCCCCCGTT

3541 CAGCCCGACC GCTGCGCCTT ATCCGGTAAC TATCGTCTTG AGTCCAACCC GGTAAGACAC

3601 GACTTATCGC CACTGGCAGC AGCCACTGGT AACAGGATTA GCAGAGCGAG GTATGTAGGC

3661 GGTGCTACAG AGTTCTTGAA GTGGTGGCCT AACTACGGCT ACACTAGAAG GACAGTATTT

3721 GGTATCTGCG CTCTGCTGAA GCCAGTTACC TTCGGAAAAA GAGTTGGTAG CTCTTGATCC

3781 GGCAAACAAA CCACCGCTGG TAGCGGTGGT TTTTTTGTTT GCAAGCAGCA GATTACGCGC

3841 AGAAAAAAAG GATCTCAAGA AGATCCTTTG ATCTTTTCTA CGGGGTCTGA CGCTCAGTGG

3901 AACGAAAACT CACGTTAAGG GATTTTGGTC ATGAGATTAT CAAAAAGGAT CTTCACCTAG

3961 ATCCTTTTAA ATTAAAAATG AAGTTTTAAA TCAATCTAAA GTATATATGA GTAAACTTGG

4021 TCTGACAGTT ACCAATGCTT AATCAGTGAG GCACCTATCT CAGCGATCTG TCTATTTCGT

4081 TCATCCATAG TTGCCTGACT CCCCGTCGTG TAGATAACTA CGATACGGGA GGGCTTACCA

4141 TCTGGCCCCA GTGCTGCAAT GATACCGCGA GACCCACGCT CACCGGCTCC AGATTTATCA

4201 GCAATAAACC AGCCAGCCGG AAGGGCCGAG CGCAGAAGTG GTCCTGCAAC TTTATCCGCC

4261 TCCATCCAGT CTATTAATTG TTGCCGGGAA GCTAGAGTAA GTAGTTCGCC AGTTAATAGT

4321 TTGCGCAACG TTGTTGCCAT TGCTACAGGC ATCGTGGTGT CACGCTCGTC GTTTGGTATG

4381 GCTTCATTCA GCTCCGGTTC CCAACGATCA AGGCGAGTTA CATGATCCCC CATGTTGTGC

4441 AAAAAAGCGG TTAGCTCCTT CGGTCCTCCG ATCGTTGTCA GAAGTAAGTT GGCCGCAGTG

4501 TTATCACTCA TGGTTATGGC AGCACTGCAT AATTCTCTTA CTGTCATGCC ATCCGTAAGA

4561 TGCTTTTCTG TGACTGGTGA GTACTCAACC AAGTCATTCT GAGAATAGTG TATGCGGCGA

4621 CCGAGTTGCT CTTGCCCGGC GTCAATACGG GATAATACCG CGCCACATAG CAGAACTTTA

4681 AAAGTGCTCA TCATTGGAAA ACGTTCTTCG GGGCGAAAAC TCTCAAGGAT CTTACCGCTG

4741 TTGAGATCCA GTTCGATGTA ACCCACTCGT GCACCCAACT GATCTTCAGC ATCTTTTACT

4801 TTCACCAGCG TTTCTGGGTG AGCAAAAACA GGAAGGCAAA ATGCCGCAAA AAAGGGAATA

4861 AGGGCGACAC GGAAATGTTG AATACTCATA CTCTTCCTTT TTCAATATTA TTGAAGCATT

4921 TATCAGGGTT ATTGTCTCAT GAGCGGATAC ATATTTGAAT GTATTTAGAA AAATAAACAA

4981 ATAGGGGTTC CGCGCACATT TCCCCGAAAA GTGCCACCTG ACGTC

//
