# Supplementary material for: Engineered transfer RNAs for suppression of premature termination codons
Source: Nat Commun. 2019 Feb 18;10:822. doi: 10.1038/s41467-019-08329-4 (PMC6379413; doi:10.1038/s41467-019-08329-4)
Supplement: Supplementary file 3 — Description of Additional Supplementary Files [file 41467_2019_8329_MOESM3_ESM.pdf]

Description of Additional Supplementary Files

File Name: Supplementary Data 1

Description: DNA sequences for ACE-tRNAs with engineered anticodons annotated 5' to 3'. Golden Gate cloning sequences are not indicated.

File Name: Supplementary Data 2

Description: Mean ± SEM and one-way ANOVA statistical test for all ACE-tRNA high-throughput screening experiments. ACE-tRNA number correlates to graphical representation of data in Supplemental Figure 2a.
